# Incident Trends, Learning Culture and Multidisciplinary Practice on a Female Acute Ward: A 12-Month Review (January–December 2025)

**DOI:** 10.1192/bjo.2026.11624

**Published:** 2026-06-30

**Authors:** Omer Malik, Sujata Sharma, Kotryna Ryan-Gillbank, Johanna Ledwaba, Neeraja Manoj

**Affiliations:** Cygnet Healthcare, London, United Kingdom

## Abstract

**Aims:**

**Background:** Female acute inpatient wards manage dynamic clinical risk, including self-harm, violence and security-related incidents. Beyond incident reduction, developing a culture of care, learning and multidisciplinary ownership is essential to achieving sustainable improvements in safety and patient experience.

**Aim:** To review incident trends over a 12-month period on a female acute ward (Hooper) at Cygnet Beckton and to describe MDT practices, learning processes and cultural factors associated with improvement and sustained low incident levels.

**Methods:**

A retrospective descriptive review of Datix-reported incidents was undertaken for January–December 2025. Incidents were analysed monthly across violence and aggression, self-harm, security incidents (including AWOL and attempted AWOL), medication-related incidents, and injury or accident events. MDT practices, learning approaches and service developments were identified through ward-level reflection and review.

**Results:**

A total of 613 incidents were recorded (monthly range 22–98).

Violence and aggression incidents totalled 182, including 85 episodes of actual physical violence, with a clear reduction across the year from 23 incidents in January to 1 incident in December.

Self-harm incidents totalled 258, primarily head banging (141) and cutting (27). Monthly self-harm peaked in May (48 incidents) and reduced to 18 incidents in December.

Security incidents remained comparatively low (47 total), including attempted AWOL/abscond (8) and AWOL (2). Medication-related incidents totalled 19, while injury and accident-related incidents totalled 47.

**Conclusion:**

MDT good practice and learning culture:

**Quality improvement approach and MDT practice:**

As part of the national accreditation-aligned QI programme, Culture of Care, Hooper ward embedded a structured culture of learning and improvement, rather than isolated incident response. MDT-led After Action Reviews were routinely undertaken following incidents, enabling systematic analysis of contributory factors, identification of improvement actions, and dissemination of lessons learned across the team.

QI interventions included care-plan-based ward rounds, shared decision-making regarding medication, patient involvement in MDT discussions (including chairing ward rounds where appropriate, chairing community meetings), co-produced safety and risk formulations, and strengthened MDT communication and escalation pathways. Learning from AARs directly informed changes to observation, engagement and care planning.

**Conclusion:**

The nationally aligned QI programme, culture of care was associated with sustained reductions in violence and self-harm on a female acute ward. Structured MDT learning, accreditation-driven standards and routine After Action Reviews supported safer, more consistent and person-centred care.

